# Adjuvant Templating Improves On-Target/Off-Target Antibody Ratio Better than Linker Addition for M2-Derived Peptide Amphiphile Micelle Vaccines

**DOI:** 10.3390/vaccines13040422

**Published:** 2025-04-17

**Authors:** Megan C. Schulte, Adam C. Boll, Natalie L. Conomos, Farnoushsadat Rezaei, Agustin T. Barcellona, Adam G. Schrum, Bret D. Ulery

**Affiliations:** 1 Department of Chemical and Biomedical Engineering, University of Missouri, Columbia, MI 65211, USAatbbtf@missouri.edu (A.T.B.);; 2 Department of Biological Sciences, University of Missouri, Columbia, MI 65211, USA; 3 Department of Molecular Microbiology and Immunology, University of Missouri, Columbia, MI 65211, USA; 4 Department of Surgery, University of Missouri, Columbia, MI 65211, USA; 5 NextGen Precision Health Institute, University of Missouri, Columbia, MI 65211, USA; 6 Materials Science & Engineering Institute, University of Missouri, Columbia, MI 65211, USA

**Keywords:** vaccine, micelle, influenza, M2, peptide amphiphile

## Abstract

Background: Peptide amphiphile micelles (PAMs) are a promising lipid-based nanotechnology currently in development for a variety of applications ranging from atherosclerosis to cancer therapy. Especially relevant for immune applications, PAMs improve trafficking through lymphatic vessels, enhance uptake by antigen-presenting cells, and inhibit the protease-mediated degradation of cargo. However, the creation of the peptide amphiphiles (PAs) necessary to induce micellization often requires modifying an immunotarget peptide with non-native moieties, which can induce the production of off-target antibodies. Methods: PAs containing different linkers between the antigen and non-native flanking regions were synthesized and physically characterized. BALB/c mice were then subcutaneously immunized on days 0 and 14 with these formulations and ELISAs were conducted on the sera collected from vaccinated mice on day 35 to evaluate antibody responses. Results: We determined that Palm_2_K-M2_2–16_-(KE)_4_ PAMs elicited off-target antibody responses and sought to avoid these unintended responses by adding linkers in between the M2_2–16_ antigen and the non-native flanking regions (i.e., Palm_2_K- and -(KE)_4_) of the PA. Most significantly, the addition of diproline linkers on either side of the M2_2–16_ antigen conferred a loss of β-sheet structure, whereas changing the method of lipid attachment from Palm_2_K- to Pam_2_CS-induced the formation of primarily spherical micelles compared to a mixture of spherical and short cylindrical micelles. Despite these morphological changes, all linker-containing PAMs still induced the production of off-target antibodies. Excitingly, however, the formulation containing a Pam_2_CS moiety (intended to mimic the adjuvanticity of the TLR2 agonist adjuvant Pam_2_CSK_4_) elicited high on-target antibody titers similar to those induced by PAMs co-delivered with Pam_2_CSK_4_. Conclusions: While the linkers tested did not completely eliminate the production of off-target antibodies elicited by the PAMs, the inclusion of a Pam_2_CS moiety both increased the amount of on-target antibodies and improved the ratio of on-target to off-target antibodies in response to the M2_2–16_ vaccine.

## 1. Introduction

Peptide amphiphile micelles (PAMs) have the potential to be highly effective drug-delivery vehicles and have been researched for a variety of applications including atherosclerosis diagnostics, cancer therapeutics, antimicrobial agents, and vaccines [1,2,3,4,5]. They offer several advantages over soluble peptides, including increasing localized concentration, site-specific trafficking, cell interaction and uptake, and cargo stability [6,7,8,9,10]. With the wide variety of bioactive peptides currently being researched, micelles also offer the ability to decrease the amount of sacrificial/non-bioactive material in the delivery vehicle compared to other nanoparticle platforms like lipid nanoparticles by attaching lipids to the bioactive peptides themselves, rather than packaging them within the nanoparticle core [11].

For vaccines in particular, PAMs have been synthesized using antigens including the J8 peptide (from Group A Streptococcus), OVA_BT_ peptide (from the model immunogen ovalbumin), and the M2_1–24_ peptide (the ectodomain of the influenza envelope M2 protein), which have been shown to enhance lymphatic vessel trafficking and uptake by antigen-presenting cells [12,13,14]. Specifically, we have established that smaller PAMs (i.e., spheres or short cylinders) are more highly immunogenic than larger PAMs (i.e., long cylinders, twines, and braids) [13]. In order to drive PAM self-assembly toward these more favorable morphologies, the moieties—such as lipids and non-native amino acid sequences—often have to be attached to the peptide antigen. In particular, attaching dipalmitoyllysine (Palm_2_K) to the N-terminus of the antigen and a (KE)_4_ block to the C-terminus of the antigen (i.e., Palm_2_K-antigen-(KE)_4_) has been shown to generate immunostimulatory spherical and short, cylindrical micelles [13].

In previous studies, we have found that Palm_2_K-M2_1–24_-(KE)_4_ PAMs, in addition to eliciting strong anti-M2_1–24_ IgG titers (when co-delivered with the adjuvant Pam_2_CSK_4_), also elicited a substantial production of off-target antibodies specific to the non-native regions of the peptide amphiphile (PA) [14]. These results were quite unexpected as these outcomes had not been previously seen using other antigens, suggesting this was a sequence-specific effect [5,12]. We have also established that, like M2_1–24_, the truncated M2_2–16_ antigen elicited strong on-target IgG titers when co-delivered with the adjuvant Pam_2_CSK_4_, both as an unmodified peptide antigen (i.e., M2_2–16_, herein referred to as Orig PMs—peptidyl micelles) and as a peptide amphiphile (PA) (i.e., Palm_2_K-M2_2–16_-(KE)_4_—referred to as Orig PAM (peptide amphiphile micelle) in this manuscript) [15]. Since the production of off-target antibodies can distract the immune system from generating a productive response, in this work, we tested whether the Palm_2_K-M2_2–16_-(KE)_4_ PAMs (like the Palm_2_K-M2_1–24_-(KE)_4_ PAMs) elicited off-target antibodies and whether various strategies could be employed to improve the on-target to off-target antibody ratio [16,17].

## 2. Materials and Methods

### 2.1. Peptide Synthesis and Purification

Pam_2_CSK_4_ was purchased from Invivogen (San Diego, CA, USA). All other peptides were made using a previously described protocol [15]. Peptides were synthesized on Sieber Amide resin (ChemPep, Wellington, FL, USA) with a Tetras Peptide Synthesizer (Louisville, KY, USA) using an Fmoc solid-phase synthesis strategy. Nα-Fmoc-L-amino acids were coupled to the resin using a ratio of 3 equivalents of amino acid, 3 equivalents hydroxybenzotriazole (HOBt), 6 equivalents N,N-diisopropylethylamine (DIPEA), and 2.7 equivalents hexafluorophosphate benzotriazole tetramethyl uranium (HBTU) in N-methylpyrrolidone (NMP) to 1-molar-equivalent resin reaction sites. Fmoc protecting groups were removed with 6% piperazine and 0.1 M HOBt in dimethylformamide (DMF). To attach dipalmitoyllysine (Palm_2_K), a non-native Fmoc-Lysine(Fmoc)-OH was attached to the N-terminus of the peptide, then both amines of the lysine were deprotected and coupled with palmitic acid using the coupling protocol described above. To attach an S-[2,3-bis(palmitoyloxy)propyl]-L-cysteine (Pam_2_C), Fmoc-Cys(Pam_2_)-OH (CAS 139573-77-6) was coupled to the N-terminal amine of the peptide using the previously described coupling procedure, then the Fmoc group was deprotected with piperazine. Biotinylation was performed by attaching a short polyethylene glycol (PEG) linker Fmoc-NH-PEG_2_-CH_2_COOH (CAS 166108-71-0), then D-biotin, to the N-terminus of the peptide, again using the same scheme. Peptides and PAs were cleaved from resin and deprotected using a cocktail of 2.5% each of water, phenol, triisopropylsilane, ethane-1,2-dithiol, and thioanisole in trifluoroacetic acid (TFA), then precipitated in diethyl ether. Peptides and PAs were purified to greater than 90% purity by high-performance liquid chromatography–mass spectroscopy (LC-MS; Beckman Coulter, Brea, CA, USA) using a gradient of water and acetonitrile (with 0.1% TFA) on either a reverse-phase C18 column for peptides or a C4 column for peptide amphiphiles. The percentage of acetonitrile at the elution of the peptides and PAs can be found in Table 1. LC-MS chromatograms and spectra for the purified peptides and PAs are provided in Figure S1.

### 2.2. Micelle Characterization

Micelle formulations were characterized by a critical micelle concentration (CMC) assay, transmission electron microscopy (TEM), and circular dichroism (CD), as previously described [15]. To determine the CMCs, peptides or PAs were serially diluted from 100 μM to 0.92 nM using a solution of 1 μM 1,6-diphenylhexatriene (DPH) in phosphate-buffered saline (PBS). After incubating in the dark for one hour, the fluorescence of the dilutions was measured at an excitation/emission of 350/428 nm using a Biotek Cytation 5 Spectrophotometer (Santa Clara, CA, USA). The CMC was established as a marked increase in fluorescence, indicating DPH entrapment within the micelle cores. More specifically, this was the intersection of logarithmic regression lines on a plot of fluorescence intensity versus peptide concentration in which one regression line was clearly below the CMC (where the slope was relatively flat) and the other above the CMC (where the slope was approximately ten times that of the first regression line).

To visualize the micelles by TEM, 100 μM peptide or PA solution in PBS was applied to a carbon-coated copper grid and incubated for 5 min, before the grid was wicked dry with filter paper. The grid was then stained with Nano-Tungsten (2% methylamine tungstate) for 5 min before the stain was wicked away with filter paper. Micrographs of the grids were captured using a JEOL JEM-1400 Transmission Electron Microscope (Tokyo, Japan) at 15,000× and 25,000× magnification. The size and aspect ratio of the micelle formulations were assessed using ImageJ Particle Size Analysis (Version 1.53e) of the TEM micrographs.

CD studies were conducted on 250 μM peptide or PA in PBS using a Jasco J-1500 Circular Dichroism Spectrometer (Oklahoma City, OK, USA). Scans were run from 190 nm to 250 nm using a 0.1 nm step size. The data were fit to reference curves of the CD of poly(lysine) and poly(glutamine) to approximate the percent character of α-helix, β-sheet, and random coil. Secondary structure averages (plus/minus one standard deviation) over three to four runs are reported.

### 2.3. Bone-Marrow-Derived Dendritic Cell (BMDC) Studies

Bone marrow was harvested from the femurs and tibias of 15-week-old BALB/c mice and processed as previously described [15]. Cells collected from the femur and tibia bone marrow were cultured in complete RPMI-1640 supplemented with 10% fetal bovine serum (FBS), 100 U/mL penicillin, 100 μg/mL streptomycin, and 50 μM granulocyte–macrophage colony-stimulating factor (GM-CSF). After 10 days, cells were plated in untreated 24-well plates and cultured with products at the dosages described in Table 2 for 24 h. Media from the treated cells were collected and frozen for further use. Cells were stained with PE-Cy7 anti-mouse CD11c (BioLegend, San Diego, CA, USA), FITC anti-mouse CD40 (BioLegend), and APC anti-mouse MHC-II (BioLegend), then fixed with 4% p-formaldehyde. Cells were analyzed using a BD LSR Fortessa Flow Cytometer (Franklin Lakes, NJ, USA) and gated according to the strategy shown in Figure S2. Cytokine secretion (TNF-α and IL-12/IL-23p40) was determined by enzyme-linked immunosorbent assays (ELISAs) using the cell media following the protocols described in the respective kits (BioLegend).

### 2.4. Vaccination Schedule

Vaccines (outlined in Table 3) consisted of 20 nmol peptide or peptide amphiphile with or without 2.22 nmol adjuvant in 100 μL of PBS. In vivo studies were conducted in accordance with protocol 32204 approved by the University of Missouri’s Animal Care and Use Committee. BALB/c mice approximately 10 weeks in age (4 females and 3–4 males per group) were subcutaneously injected in the nape of the neck with the vaccines. Immunizations were given on days 0 and 21, with blood collected via cardiac puncture after CO_2_ asphyxiation on day 35.

### 2.5. Serum Enzyme-Linked Immunosorbent Assay (ELISA)

Serum was separated from blood samples by centrifuging the blood at 9400× *g* for 10 min and collecting the supernatant. Serum was frozen at −80 °C until future use. ELISAs were conducted to quantify the antibody content, as previously described [15]. Briefly, 1.69 μM coating antigen (or 4 μg/mL streptavidin) in carbonate buffer was incubated in Maxisorp ELISA plates (Thermo Fisher Scientific, Waltham, MA, USA) at 4 °C overnight. For streptavidin plates, the wells were then washed three times with 0.05% Tween-20 in PBS, after which 1.69 μM of biotinylated antigen in carbonate buffer was incubated in the wells for 1 h. All plates were then blocked for 1 h with assay diluent (10% fetal bovine serum (FBS) in PBS) followed by incubation at 4 °C overnight with murine serum samples that were serially diluted 2-fold in assay diluent 21 times from a 100-fold to a 209,715,200-fold dilution. The next day, plates were incubated with a 1:3000 dilution of goat anti-mouse IgG-HRP (Invitrogen, Waltham, MA, USA) secondary antibody in assay diluent for 1 h, then with TMB (3,3′,5,5′ tetramethylbenzidine, BioLegend) substrate for 30 min. The absorbance of the wells was measured at 650 nm using a Biotek Cytation 5 Spectrophotometer. Samples were normalized across plates by subtracting the absorbance of assay-diluent-only wells from the absorbance of each serum-containing well. Antibody titers were calculated as the lowest serum dilution with an absorbance at least twice the average absorbance of the serum samples from PBS-vaccinated mice at a given serum dilution.

### 2.6. Statistics

A one-way analysis of variance (ANOVA) followed by Tukey’s Honestly Significant Difference (HSD) test was performed using GraphPad Prism software (Version 7.02). Within a graph, groups that possess different letters have statically significant differences in means (*p* ≤ 0.05) whereas those that have the same letter have similar means (*p* > 0.05).

## 3. Results

### 3.1. Palm_2_K-M2_2–16_-(KE)_4_ Peptide Amphiphile Micelles Elicited Off-Target Antibody Production

**Figure 1:**
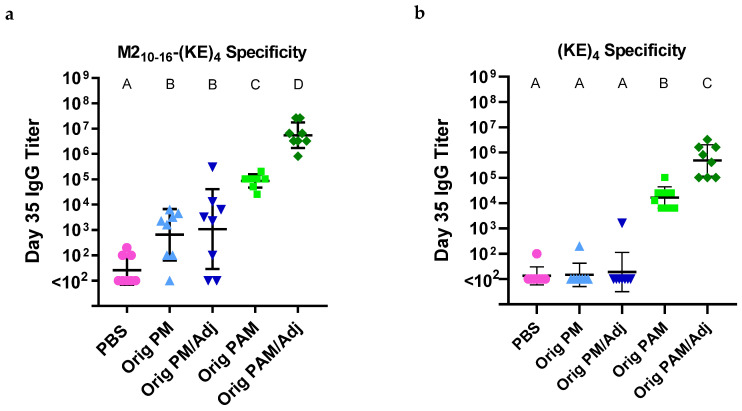
PAMs elicited high IgG titers specific to M2_10–16_-(KE)_4_ and (KE)_4_: (**a**) Unmodified M2_2–16_ (i.e., Orig PM and Orig PM/Adj) elicited titers specific to M2_10–16_-(KE)_4_ above baseline, regardless of the inclusion of Pam_2_CSK_4_ (Adj). Palm_2_K-M2_2–16_-(KE)_4_ (i.e., Orig PAMs), especially when co-delivered with adjuvant, produced titers significantly higher than Orig PM groups. (**b**) Only Orig PAM-containing groups elicited titers specific to (KE)_4_, with incorporated adjuvant further boosting this response. Within a graph, groups that possess different letters have statistically significant differences in means (*p* ≤ 0.05) whereas those that possess the same letter have similar means (*p* > 0.05). Statistical groups between graphs are unrelated.

To test whether Palm_2_K-M2_2–16_-(KE)_4_ PAMs induced off-target antibodies, mice were vaccinated with Orig PMs (with or without Pam_2_CSK_4_ (i.e., Adj)) or Orig PAMs (with or without Adj) on days 0 and 21. Blood was collected on day 35 and used in ELISAs to quantify the antibody response to the vaccine formulations. ELISAs were conducted to evaluate serum antibody specificity to the C-terminus of the Palm_2_K-M2_2–16_-(KE)_4_ PA, specifically using coating antigens of M2_10–16_-(KE)_4_ (Figure 1a) and (KE)_4_ (Figure 1b). In Figure 1a, M2_2–16_ (i.e., Orig PM) and M2_2–16_/Pam_2_CSK_4_ (i.e., Orig PM/Adj) induced moderate anti-M2_10–16_-(KE)_4_ IgG titers, whereas Orig PAM and Orig PAM/Adj induced higher and the highest titers, respectively. Results were even more stark in Figure 1b, in which there were expectedly no detectable anti-(KE)_4_ titers in the vaccine groups containing Orig PMs but there were strong titers in both Orig PAM-containing vaccine groups, confirming the presence of off-target antibodies.

### 3.2. Proline–Proline and Pam_2_CS Moieties Induced Slight Changes to the Physical Properties of M2_2–16_-Containing Peptide Amphiphile Micelles

While we have not yet determined whether the off-target antibodies negatively impact PAM vaccine efficacy, we sought to improve the on-target to off-target antibody ratio elicited by PAMs. Based on the data presented in Figure 1 and previous work using the M2_1–24_ antigen, it seemed that antibodies were being produced with partial specificity to both the M2 antigen and the non-native flanking regions (i.e., Palm_2_K and (KE)_4_) [14]. Thus, we hypothesized that linkers could be used to alter the PA conformation and/or add distance to prevent antibodies from simultaneously binding to both the antigen and the (KE)_4_ block.

**Figure 2:**
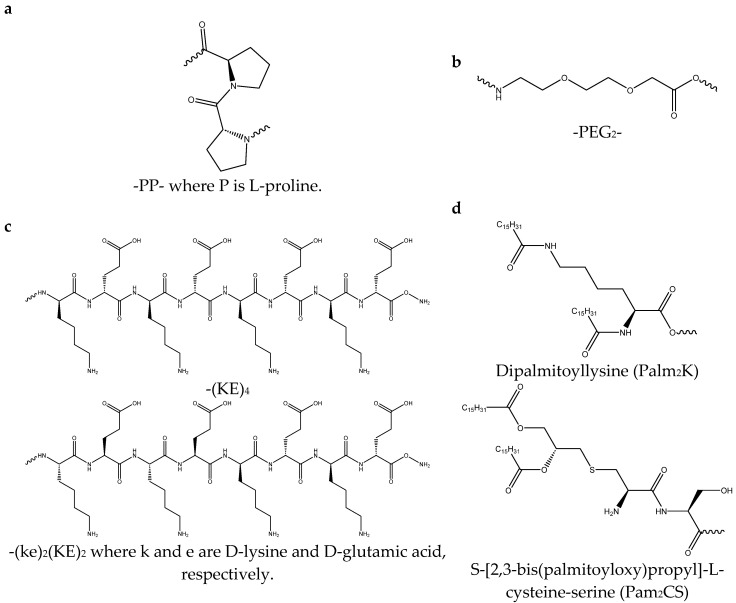
Linkers were inserted between the non-native moiety and the antigen on either side of the M2_2–16_ peptide. In PP PAMs and PEG_2_ PAMs, (**a**) PP and (**b**) PEG_2_ were added to both termini of M2_2–16_, respectively. (**c**) In ke PAMs, the first four residues of the charge block were inverted from L-amino acids ((KE)_4_) to D-amino acids ((ke)_2_(KE)_2_). (**d**) In P_2_CS PAMs, lipids were attached to the thiol on the side chain of the cysteine (Pam_2_CS) via a glycerol molecule instead of the α- and ε-amines of the lysine (as in Palm_2_K).

Several different linkers were tested to investigate how they changed micelle morphology and secondary structure as well as the subsequent antibody response. The specific formulations used are listed in Table 4. Double proline linkers (Figure 2a) on both sides of the antigen were used to disrupt the PA secondary structure because of the kinking that prolines cause due to their secondary α-amine [18,19]. PEG_2_ linkers (Figure 2b) were used to disrupt the amide backbone of the peptide and add distance and flexibility between the antigen and non-native modifications [20]. The incorporation of D-amino acids into the (KE)_4_ charge block (instead of the usual L-amino acids) was another approach to disrupt the secondary structure (Figure 2c) [21,22,23,24,25]. Lastly, the method of lipid attachment to the antigen was changed (Figure 2d) by replacing Palm_2_K (in which the lipids were attached by amide linkages to the α- and ε-amines of the lysine) with Pam_2_CS (in which both lipids were attached via a glycerol to the thiol of the cysteine). In multiple previous vaccine studies using a variety of antigens, the conjugation of the Pam_2_C moiety to a peptide antigen has been shown to elicit strong immune responses [26,27,28,29,30,31]. Specifically, the templation of Pam_2_CS to the N-terminus of the antigen could potentially allow for the lipids of the resulting PA to impart adjuvant activity (as it mimics the structure of the TLR2 agonist Pam_2_CSK_4_) in addition to influencing PA off-target antibody production [5,32]. The additional serine between Pam_2_C and M2_2–16_ was included because it has been shown to be important for TLR2 activation [32].

**Figure 3:**
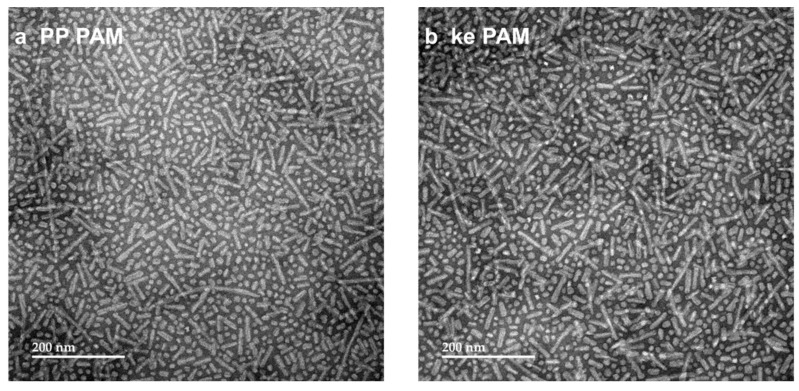

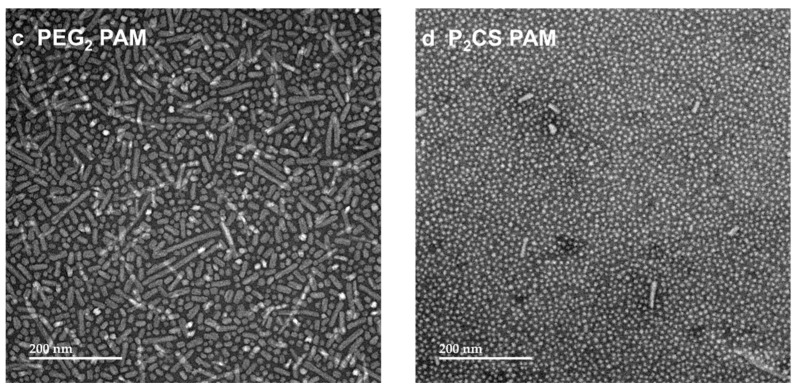
TEM of M2_2–16_ PAMs with linkers show that all PAs formed small micelles. (**a**) PP PAMs, (**b**) ke PAMs, and (**c**) PEG_2_ PAMs were all a similar mix of spherical and short cylindrical micelles. (**d**) P_2_CS PAMs were primarily spherical micelles.

The new formulations were synthesized and purified by LC-MS (Figure S1). The PAs were characterized by CMC, TEM, and CD. CMC assessments showed that all new PAs formed micelles, with the PP PAMs and P_2_CS PAMs having slightly higher CMCs compared to Orig PAMs and the other formulations (Table 5). CMC curves are displayed in Figure S3. All formulations were micellized at sufficiently low enough concentrations to ensure sustained micelle presence in vivo [33]. TEM micrographs (Figure 3) showed that, like the original formulation Palm_2_K-M2_2–16_-(KE)_4_ (Orig PAM), all of the PAs formed small spherical and/or short cylindrical micelles (with average maximum diameters of 22–24 nm, as seen in Table 6). P_2_CS PAMs were the only PAMs with noticeably different morphology from Orig PAMs, yielding almost entirely spherical micelles alone (Figure 3d) and an average maximum diameter of 11 ± 4 nm (Table 6).

**Figure 4:**
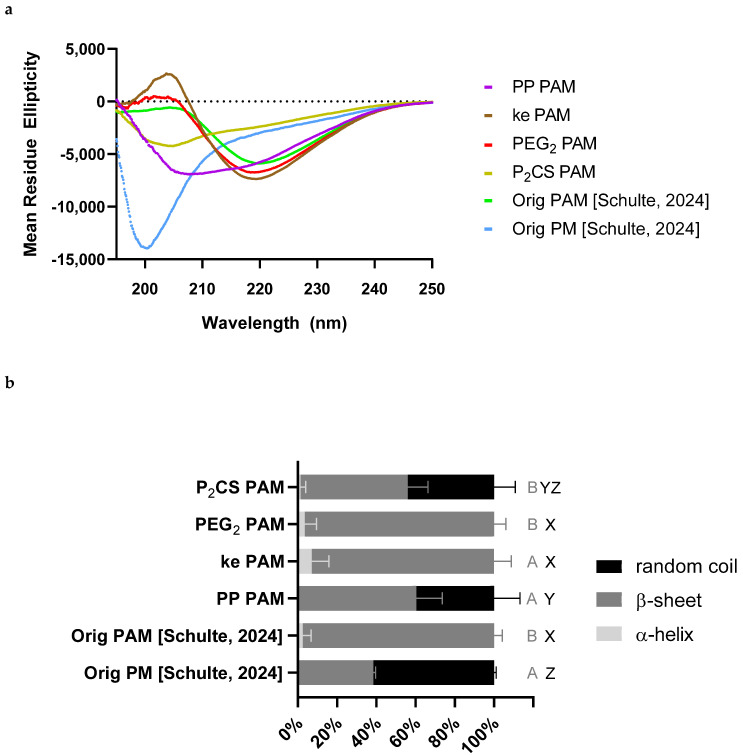
Only peptide amphiphiles with the proline–proline linkers or Pam_2_C templation had different secondary structures from the original PAM formulation [15]. (**a**) The CD spectra of the linker-containing PAMs show differences in the location of the minima (from Orig PAM) for only PP and P_2_CS PAMs at approximately 208 and 205 nm, respectively, which indicated a change in secondary structure for those formulations. (**b**) PEG_2_ and ke PAMs were nearly entirely β-sheet, like the Orig PAMs. PP and P_2_CS PAMs exhibited less β-sheet and more random coil character, more similar to Orig PMs. Groups that possess different letters have statistically significant differences in means (*p* ≤ 0.05) whereas those that possess the same letter have similar means (*p* > 0.05). Dark gray and black letters signify statistical groups based on β-sheet and random coil percentages, respectively. There were no statistical differences when comparing the α-helix content of any of the formulations.

To further characterize the PAMs, CD was performed on all formulations (Figure 4a) to determine whether any of the linkers produced a substantial change in the secondary structure from the Orig PAMs (i.e., Palm_2_K-M2_2–16_-(KE)_4_). The PA’s secondary structure was estimated from the CD spectra using known reference curves (Figure 4b). All micelle formulations showed predominantly β-sheet character and minimal α-helical behavior (10% or less), with the secondary structures of ke PAMs and PEG_2_ PAMs closely aligning with that of the Orig PAMs. Lipidating the peptide using Pam_2_Cys (i.e., in P_2_CS PAMs) and using double-proline spacers between the antigen and flanking regions of the peptide (i.e., in PP PAMs) caused secondary structure changes that reduced the β-sheet presence and increased random coil content. The addition of PEG_2_ linkers between the antigen and non-native regions (i.e., in PEG_2_ PAMs and ke PAMs) did not introduce secondary structure changes.

### 3.3. Bone-Marrow-Derived Dendritic Cells Were Activated by P_2_CS PAMs

**Figure 5:**
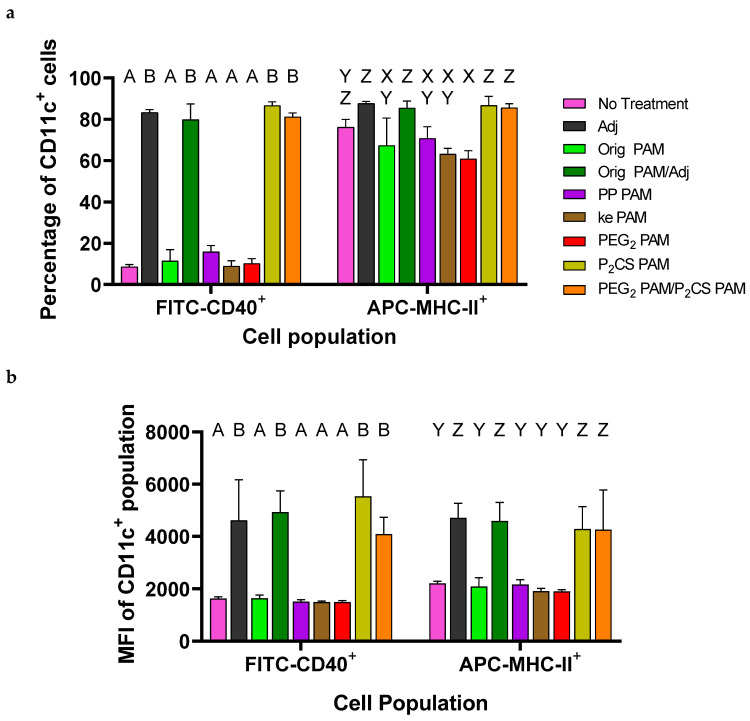

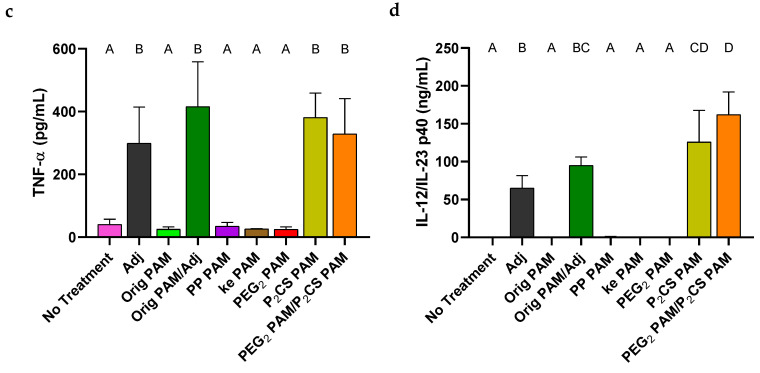
Treatment groups containing Adj or P_2_CS PAMs effectively activated BMDCs. (**a**) The percentage of BMDCs expressing elevated levels of CD40 (and to a lesser extent MHC-II) increased in treatment groups containing Adj or P_2_CS PAMs. (**b**) The MFI in CD40^+^CD11c^+^ and MHC-II^+^CD11c^+^ cells was higher when treated with formulations containing either adjuvant. (**c**) TNF-α secretion was elevated in cells treated with any formulation with Adj or P_2_CS PAMs. (**d**) IL-12/IL-23 p40 secretion increased in BMDC treatment groups containing adjuvant with this being further enhanced by the presence of P_2_CS PAMs. Within a graph, groups that possess different letters have statistically significant differences in means (*p* ≤ 0.05) whereas those that possess the same letter have similar means (*p* > 0.05). Statistical groups in different graphs are unrelated.

To evaluate whether changes in micelle morphology would influence innate immune responses against the linker-containing PAMs when compared to Orig PAMs, BMDCs were cultured and treated with the various formulations. After 24 h, the media were removed from the cells and saved to evaluate cytokine secretion using ELISAs. Cells were stained with PE-Cy7 anti-mouse CD11c, FITC anti-mouse CD40, and APC anti-mouse MHC-II antibodies and processed for flow cytometry. BMDC activation was detected by upregulated CD40 and/or MHC-II expression on the CD11c^+^ cells. The percentage of CD11c^+^ cells expressing elevated levels of CD40 was highest in treatment groups containing Adj (i.e., Pam_2_CSK_4_) or P_2_CS PAMs (Figure 5a). To a much lesser extent, MHC-II^+^CD11c^+^ cell percentages were also elevated in adjuvant-containing groups but without statistical significance as the No Treatment group possessed a high percentage of these cells already. Further evidence of BMDC activation by adjuvants was seen by the increased FITC median fluorescence intensity (MFI) in CD40^+^CD11c^+^ cells and increased APC MFI in MHC-II^+^CD11c^+^ cells (Figure 5b). Flow cytometry data did not indicate significant BMDC activation in the PP PAM, ke PAM, or PEG_2_ PAM treatment groups in the absence of an adjuvant.

To further determine BMDC activation, sandwich ELISAs were conducted using the media collected from the treated BMDCs. These assays quantified pro-inflammatory cytokine secretion—in particular TNF-α (Figure 5c) and IL-12/IL-23 p40 (Figure 5d)—as these two have been commonly shown to be cell signaling markers of activated BMDCs [34,35,36]. In reasonable agreement with the flow cytometry data, increased secretions of TNF-α and IL-12/IL-23 p40 were seen in cells treated with adjuvant (again, either Pam_2_CSK_4_ or P_2_CS PAMs), while groups treated with PAMs without adjuvant exhibited similar levels of TNF-α and IL-12/IL-23 p40 secretion to the No Treatment group.

### 3.4. Linker-Containing Peptide Amphiphile Micelles Elicited Off-Target IgG Antibodies but P_2_CS PAMs Also Elicited Strong On-Target Titers

**Figure 6:**
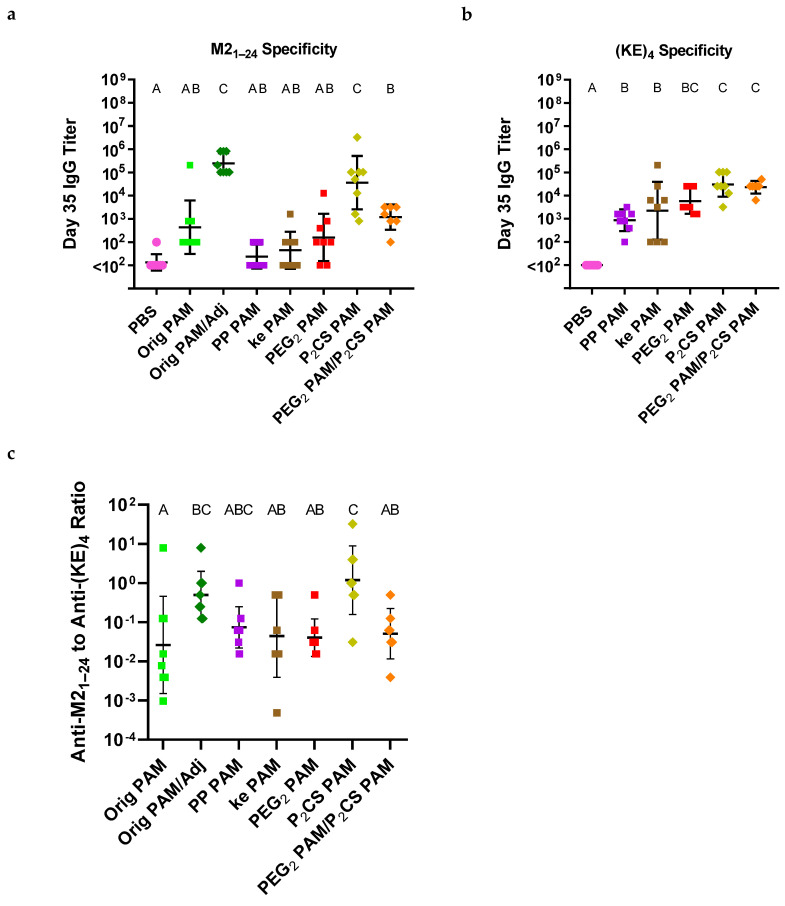
Of the linker-containing PAMs, only P_2_CS PAMs elicited high on-target antibody titers, while all linker-containing PAMs elicited off-target antibodies against the (KE)_4_ region. (**a**) Linker-containing PAMs (with the exception of P_2_CS PAM) generated low on-target (anti-M2_1–24_) antibody titers, similar to Orig PAM alone. P_2_CS PAM-containing vaccine groups elicited higher titers, with similar titers between P_2_CS PAM and Orig PAM/Adj. (**b**) All linker-containing PAMs elicited roughly similar levels of off-target anti-(KE)_4_ IgG titers, although P_2_CS PAM-containing groups elicited titers statistically higher than PP PAM and ke PAM. (**c**) P_2_CS PAMs and Orig PAM/Adj had the highest ratios of on-target to off-target antibody titers compared to the other formulations. Groups that possess different letters have statistically significant differences in means (*p* ≤ 0.05) whereas those that possess the same letter have similar means (*p* > 0.05). Statistical groups between graphs are unrelated.

To evaluate the immunogenicity of the linker-containing PAMs in vivo, mice were administered two doses of PAM vaccines (on days 0 and 21), using the same protocol described above. The vaccine groups can be found in Table 3. In addition to testing the in vivo immunogenicity of each linker-containing PAM individually, an additional group was included to test the adjuvant effect of P_2_CS PAMs at a dose equivalent to the dose of Pam_2_CSK_4_ in the Orig PAM/Adj vaccine group (i.e., 2.22 nmol P_2_CS PAMs co-delivered with 20 nmol PEG_2_ PAMs). Blood was collected on day 35 and used in ELISAs to evaluate antibody content. Serum antibody titers specific to M2_1–24_ (the complete ectodomain of the influenza M2 protein) were quantified to measure on-target antibody specificity (Figure 6a). Excitingly, P_2_CS PAM-vaccinated mice exhibited high on-target IgG titers similar to that in Orig PAMs/Adj-vaccinated mice. The remaining linker-containing PAMs (including the PEG_2_ PAM/P_2_CS PAM group) elicited significantly weaker on-target titers against M2_1–24_, on par or below the titers of non-adjuvanted Orig PAMs. To test whether these PAMs were also inducing off-target antibodies, an ELISA testing antibody specificity against (KE)_4_ was conducted (Figure 6b). This test confirmed the existence of somewhat comparable amounts of off-target antibodies among all linker-containing PA formulations, although anti-(KE)_4_ titers were statistically significantly higher among P_2_CS PAM-containing vaccine groups than in the PP PAM and ke PAM groups. Interestingly, when the ratios of on-target to off-target antibody titers were calculated for each animal using anti-M2_1–24_ and anti-(KE)_4_ titers, these were found to be more favorably skewed toward on-target antibody production for adjuvant-containing vaccine groups, with P_2_CS PAMs having a slightly higher ratio than Orig PAM/Adj (Figure 6c).

### 3.5. IgG Titers Were More Dependent on the Sequence than the Attachment Method of the Coating Antigen

While it is evident that significant quantities of off-target antibodies were elicited by the linker-containing PAMs, we sought to determine if there were any additional explanations for the lower-than-expected on-target antibody titers in the PAM groups relative to the Orig PM/Adj (i.e., M2_2–16_/Pam_2_CSK_4_) group. We hypothesized that, especially given the commonality of conformational B-cell epitopes [37], the currently used method of attaching the coating antigen to the ELISA plate (i.e., passive adsorption) could have a confounding effect on the antibody titers detected. This would be especially true if the coating antigen did not favorably present the target epitope for antibody binding, something that has been previously shown in the literature [38,39,40,41,42].

**Figure 7:**
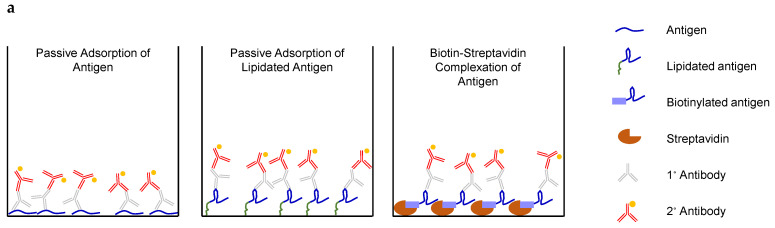

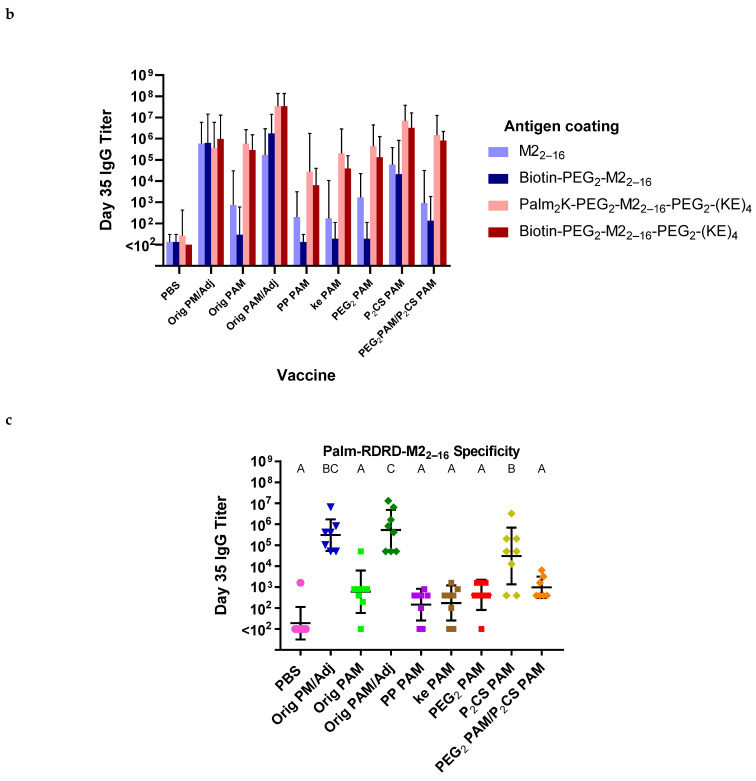
Coating antigens containing C-terminal (KE)_4_ blocks captured high titers in PAM vaccine groups. (**a**) Passive adsorption of a peptidyl or lipidated coating antigen directly to the plate surface (left and middle, respectively) or complexation of a biotinylated coating antigen to streptavidin (right) might change recognizability of the coating antigen by primary antibodies. (**b**) Unbiotinylated and biotinylated coating analogs (i.e., light and dark bars of the same color) captured comparable titers to each other. Orig PM-containing group titers were similar regardless of coating antigen. PAMs produced higher titers (than Orig PM/Adj) specific to the coating antigens containing non-native C-terminal modifications (i.e., (KE)_4_). Statistical groups were created using one-way ANOVA and Tukey’s HSD test to evaluate differences between vaccine groups for a given coating antigen (Table S1) and differences between coating antigens for a given vaccine group (Table S2). (**c**) An ELISA using Palm-RDRD-M2_2–16_ as the coating antigen showed high titers in groups containing either Pam_2_CSK_4_ or 100% P_2_CS PAM. Other PAMs without Pam_2_CSK_4_ were not statistically above baseline. Groups that possess different letters have statistically significant differences in means (*p* ≤ 0.05) whereas those that possess the same letter have similar means (*p* > 0.05).

To investigate this possible effect, a few immobilization approaches were tested (Figure 7a). As has been used previously, the first strategy was passive adsorption of the unmodified M2_2–16_ antigen, in which the coating antigen associates with the high-binding surface of a Maxisorp plate via hydrophobic and other non-covalent forces. In this case, the truncated M2_2–16_ antigen was used, rather than the previously employed M2_1–24_ coating antigen, to allow for more equivalent comparisons to the other coating approaches. The second strategy involved passive adsorption of the Palm_2_K-PEG_2_-M2_2–16_-PEG_2_-(KE)_4_ PA to the plate to test whether the lipids—rather than the peptidyl portion of the PA—preferentially bind to the hydrophobic plate surface, thus potentially affecting antigen display. This approach also allowed us to test for the presence of any N-terminal-specific off-target antibodies. The last strategy was streptavidin complexation with biotinylated analogs of either M2_2–16_ peptide or Palm_2_K-PEG_2_-M2_2–16_-PEG_2_-(KE)_4_ PA. To achieve this, streptavidin was first adsorbed to the plate surface, then the biotinylated “coating” antigen (either Biotin-PEG_2_-M2_2–16_ or Biotin-PEG_2_-M2_2–16_-PEG_2_-(KE)_4_, the analog to the PA coating antigen) added to initiate complexation. The rationale for biotin–streptavidin complexation was that this method could allow the antigen more conformational freedom due to its single anchoring point compared to the two-dimensional constraints of the unspecific passive adsorption method.

Interestingly, the IgG titers against the biotinylated coating antigens (Figure 7b) largely aligned with those of their analogous unbiotinylated coating antigens (i.e., for most cases, the light and dark blue bars were similar to each other and the light and dark red bars aligned) for each given vaccine group. For the non-PAM vaccine groups (i.e., PBS and Orig PM/Adj), IgG titers were also very consistent across all four coating antigens tested. The titers for each PAM vaccine group, however, were significantly higher for the coating antigen Palm_2_K-PEG_2_-M2_2–16_-PEG_2_-(KE)_4_ (light red bars) and its biotinylated analog (i.e., Biotin-PEG_2_-M2_2–16_-PEG_2_-(KE)_4_, dark red bars) than for the coating antigen M2_2–16_ (light blue bars) and its biotinylated analog (i.e., Biotin-PEG_2_-M2_2–16_, dark blue bars). For coating antigens M2_2–16_ and Biotin-PEG_2_-M2_2–16_, the presence of adjuvant in the vaccine was the biggest predictor of high titers, as seen for the coating antigen M2_1–24_ in Figure 6a. Notably, titers induced by PAM vaccine groups without an adjuvant (i.e., Orig PAM, PP PAM, ke PAM, and PEG_2_ PAM) were lower than Orig PM/Adj for these two coating antigens. However, for the PA coating antigen and its biotinylated analog, PAM vaccines (with or without adjuvant) elicited comparable or higher titers than Orig PM/Adj.

To further probe this effect, a final ELISA was conducted, employing the passive adsorption method of attachment, using the coating antigen Palm-RDRD-M2_2–16_ (Figure 7c). This PA was chosen to evaluate antibody binding to a lipidated M2_2–16_, while eliminating the non-native peptidyl modifications used in Palm_2_K-M2_2–16_-(KE)_4_ and the linker-containing PAs to avoid capturing off-target antibodies. The RDRD repeat was specifically chosen to improve solubility of the PA. IgG titers captured by Palm-RDRD-M2_2–16_ largely aligned with trends seen in Figure 6a (using M2_1–24_ as the coating antigen) and Figure 7b (using M2_2–16_ or Biotin-PEG_2_-M2_2–16_ as the coating antigen), namely that titers were highest among vaccine groups containing adjuvant (i.e., Pam_2_CSK_4_) or the higher concentration of P_2_CS PAMs.

## 4. Discussion

In this work, we showed that Palm_2_K-M2_2–16_-(KE)_4_ PAMs, like the previously studied Palm_2_K-M2_1–24_-(KE)_4_ PAMs, induced off-target antibody production (Figure 1); however, we were able to significantly improve the on-target to off-target antibody ratio by templating a Pam_2_CS moiety to the N-terminus of the M2 antigen, as shown with P_2_CS PAMs [14]. That being said, we have yet to determine whether this off-target antibody production is actually detrimental to the efficacy of these PAM vaccines. However, given that in other studies there was an enhancement in IgG titer from unmodified OVA_BT_ or J8 peptides to their analogous PAMs but not in our studies using M2e antigens (i.e., M2_2–16_ or M2_1–24_ peptide), it is reasonable to suggest that Palm_2_K-M2_2–16 or 1–24_-(KE)_4_ PAMs could induce higher on-target titers in the absence of immune distraction [5,12,15].

As such, we tested different strategies to prevent the production of off-target antibodies by adding linkers between the antigen and non-native moieties of the PA. Given that the general configuration of Palm_2_K-antigen-(KE)_4_ was preserved in the linker-containing formulations (which has been previously shown in multiple cases to elicit small micelles), it is not surprising that all of the linker-containing PAs formed small spherical and/or cylindrical micelles, as shown by TEM in Figure 3 [14,43,44]. The slightly different lipid “orientation” from the Pam_2_CS moiety in the P_2_CS PAMs compared to the other PAMs (i.e., those lipidated with dipalmitoyllysine) seemed to have the most significant effect on micelle shape as Pam_2_CS-M2_2–16_-PEG_2_-(KE)_4_ was the only PA that formed almost entirely spherical micelles alone. This aligned with the mostly spherical morphology that has been previously observed in Pam_2_CSK_4_-only micelles [15,45]. More significant differences between the formulations were seen in the peptide secondary structure (Figure 4). Specifically, P_2_CS PAMs possessed more disordered random coil structure content than Orig PAMs, likely due to their increased sphericity, as was observed with Orig PMs (i.e., M2_2–16_ peptide) and has also been noted in the literature [3,15,46]. The increased random coil content in the P_2_CS PAMs could also likely be attributed to the Pam_2_CS moiety as the CD of Pam_2_CSK_4_ (Figure S4) showed a secondary structure composition of 81.6% random coil and 18.4% β-sheet, which agrees with previously published results [45]. In the PP PAMs, the reduced percentage of β-sheet content could be attributed to the addition of the proline–proline linkers as this same effect has been shown when substituting a serine for a proline in Alzheimer’s disease-associated Tau protein [19]. As for the lack of conformational changes in the PEG_2_-containing PAMs, although polyethylene glycols are known for their ability to increase the flexibility of polymers, PEG_2_ is only a dimer of ethylene glycol, which is likely too short to induce any significant changes in the peptide secondary structure [20]. Finally, the lack of any structural changes in the ke PAM was unexpected due to the frequent use of D-amino acids in the literature to induce functional or structural changes in peptides [21,22,23,24,25]. Further, D-amino acids have been used specifically for the disruption of β-sheets and affecting antibody recognition, thus it is likely that the location of the substitutions or the number of D-amino acids for the studied product was insufficient to induce structural changes [21,24].

The in vitro bioactivity of the linker-containing PAMs (Figure 5) agreed with previous work in that PAMs alone were not strongly immunogenic but rather, the presence of adjuvant was the primary driver of BMDC activation [5,15]. The use of most PA linkers did not induce BMDC activation besides P_2_CS PAMs, which were excitingly found to be immunogenic. This result suggested that the P_2_CS PAMs possessed a functional adjuvant, most likely by maintaining the TLR2/TLR6-agonist behavior found with Pam_2_CSK_4_. Activation of these TLRs together triggers a signal cascade that stimulates the secretion of pro-inflammatory cytokines including TNF-α, IL-12, and IL-23, as well as the increased expression of MHC-II and CD40—among other maturation markers—in BMDCs [36,47,48,49]. This aligns well with the signs of activation observed with the P_2_CS PAM treatment. The adjuvant activity of P_2_CS PAMs was especially notable given that our group has previously shown that templating the OVA_BT_ antigen with just Pam_2_C (i.e., Pam_2_C-OVA_BT_-(KE)_4_) was insufficient to increase the immunogenicity of the PA [5]. The results here lend credence to the suggestion that the serine residue is indeed important for TLR2 activation by Pam_2_C, as has been shown in the literature [32,50].

The in vivo immunogenicity of the PAMs aligned well with the BMDC results. Distinctly, high on-target IgG titers in P_2_CS PAM-vaccinated mice provided additional evidence that the templation of Pam_2_CS to the N-terminus of the M2_2–16_ antigen allowed the PAM to act both as an adjuvant and an antigen (Figure 6a). The similar anti-M2_1–24_ titers between P_2_CS PAMs and Orig PAMs/Adj, despite the higher dosage of the Pam_2_CS moiety in the P_2_CS PAMs (i.e., 20 nmol vs. 2.22 nmol, respectively), suggested however that unmodified Pam_2_CSK_4_ was a more potent adjuvant. The lower anti-M2_1–24_ titers in the PEG_2_ PAM/P_2_CS PAM vaccine, despite equimolar amounts of Pam_2_CS moiety to the Orig PAM/Adj vaccine, further confirmed this. While the K_4_ region of Pam_2_CSK_4_ does not play a primary role in TLR2/TLR6 activation by Pam_2_CSK_4_, this region does form weak ionic and hydrogen bonds with the TLR so it is possible that the absence of a cationic K_4_ region resulted in some loss of adjuvant activity in the P_2_CS PAMs (compared to Orig PAMs/Adj) [32,51]. Another possibility is that steric hindrance due to the presence of the M2_2–16_ antigen is affecting the binding of the P_2_CS PAMs to the TLRs.

As seen from the anti-(KE)_4_ ELISA results (Figure 6b), off-target antibody production still occurred in response to vaccination with all of the linker-containing PAMs. Nonetheless, the fact that the P_2_CS PAMs elicited a relatively comparable anti-(KE)_4_ titer—although statistically distinct—to the other linker-containing PAMs while displaying a much stronger on-target anti-M2_1–24_ titer is worth noting. When comparing the ratio of on-target to off-target antibodies between all the PAM groups, Orig PAM/Adj, and especially P_2_CS PAMs, had more favorable ratios skewed toward relatively similar on-target to off-target antibody production. In contrast, all other PAM groups possessed a ratio well below one, indicating off-target antibodies were produced in much higher quantities than on-target antibodies.

In an effort to ascertain whether the observed antibody titers were solely due to off-target antibody production, we found that differences in PAM vaccine titers between the ELISA coating antigens aligned more closely with the presence or absence of the (KE)_4_ region in the coating antigen than their attachment method (Figure 7b). The consistency in titers between the coating antigens (i.e., M2_2–16_ or Palm_2_K-PEG_2_-M2_2–16_-PEG_2_-(KE)_4_) and their biotinylated analogs suggests the validity of the streptavidin–biotin coating strategy as an alternative to the passive adsorption coating method. This technique would be quite beneficial for when passive adsorption prevents antibody access to its cognate epitope within the coating peptide/protein. Furthermore, the similarity in titers between the PA coating antigen and its biotinylated analog demonstrated the lack of off-target antibodies specific to the N-terminus due to the fact that the lipidated PA coating antigen did not capture higher titers relative to the M2_2–16_ coating antigen. Further investigation into this behavior demonstrated that a coating antigen of Palm-RDRD-M2_2–16_ produced results that aligned very closely with the M2_1–24_ and M2_2–16_ ELISAs (Figure 7c), despite the presence of a palmitoyl group on the N-terminus of the coating antigen. Thus, any hypothetical conformational constraints on the coating antigen did not appreciably impact antibody titers. This provided additional evidence that titer differences between the vaccine groups were indeed due to the presence of antibodies specific to a non-antigenic component of the PA (i.e., (KE)_4_). It is also important to note, however, that the Maxisorp ELISA plates used in this study were sufficiently amphiphilic to favorably bind both peptides and peptide amphiphiles—without a strong preference for lipid binding—but plates using other surface treatments not optimized for peptide adsorption could elicit different results [52,53,54].

## 5. Conclusions

While Palm_2_K-M2_2–16_-(KE)_4_ PAMs elicited strong immune responses in vitro and in vivo when co-delivered with an adjuvant, the non-native (KE)_4_ charge block induced substantial production of off-target antibodies specific to the (KE)_4_ peptide. PA formulations containing proline–proline linkers flanking the M2_2–16_ antigen or an N-terminal Pam_2_CS moiety induced some changes to their critical micelle concentration, morphology, and secondary structure but all formulations tested still induced a significant off-target antibody response against the (KE)_4_ region of the PAs. While the linkers tested did not abrogate off-target antibody production, we found that templation of the M2_2–16_ peptide by a Pam_2_CS moiety to yielded a PA (i.e., Pam_2_CS-M2_2–16_-PEG_2_-(KE)_4_) that mimics the adjuvant activity of Pam_2_CSK_4_, which resulted in desirable BMDC activation and elevated antibody titers with a more favorable on-target to off-target ratio. The covalent attachment of an adjuvant and antigen could be especially beneficial for applications where localized co-delivery of both components is critical, such as with in situ vaccines.

## Figures and Tables

**Table 1:** Percent acetonitrile at peptide and peptide amphiphile elution time.

Abbreviation	Peptide/PA	% Acetonitrile at Elution
Orig PM	M2_2–16_	35%
Orig PAM	Palm_2_K-M2_2–16_-(KE)_4_	65%
	M2_10–16_-(KE)_4_	25%
	(KE)_4_	5%
PP PAM	Palm_2_K-PP-M2_2–16_-PP-(KE)_4_	65%
ke PAM	Palm_2_K-PEG_2_-M2_2–16_-(ke)_2_(KE)_2_	65%
PEG_2_ PAM	Palm_2_K-PEG_2_-M2_2–16_-PEG_2_-(KE)_4_	70%
P_2_CS PAM	Pam_2_CS-M2_2–16_-PEG_2_-(KE)_4_	65%
	M2_1–24_	35%
	Biotin-PEG_2_-M2_2–16_	30%
	Biotin-PEG_2_-M2_2–16_-PEG_2_-(KE)_4_	30%
	Palm-RDRD-M2_2–16_	40%

Note: M2_2–16_ is SLLTEVETPIRNEWG and M2_1–24_ is MSLLTEVETPIRNEWGCRCNDSSD; Palm_2_K is dipalmitoyllysine. Standard one-letter designations are used for L-amino acids. (KE)_x_ or (ke)_x_ is a repeat of L-lysine-L-glutamic acid or D-lysine-D-glutamic acid, respectively, x times; PEG_2_ is a polyethylene glycol linker made from Fmoc-NH-PEG_2_-CH_2_COOH; and Pam_2_C is S-[2,3-bis(palmitoyloxy)propyl]-L-cysteine.

**Table 2:** Vaccine dosages for BMDC activation study.

Experimental Group	Treatment
No Treatment	n/a
Adj	0.2 μM Pam_2_CSK_4_
Orig PAM	1.8 μM Palm_2_K-M2_2–16_-(KE)_4_
Orig PAM/Adj	1.8 μM Palm_2_K-M2_2–16_-(KE)_4_ and 0.2 μM Pam_2_CSK_4_
PP PAM	1.8 μM Palm_2_K-PP-M2_2–16_-PP-(KE)_4_
ke PAM	1.8 μM Palm_2_K-PEG_2_-M2_2–16_-(ke)_2_(KE)_2_
PEG_2_ PAM	1.8 μM Palm_2_K-PEG_2_-M2_2–16_-PEG_2_-(KE)_4_
P_2_CS PAM	1.8 μM Pam_2_CS-M2_2–16_-PEG_2_-(KE)_4_
PEG_2_ PAM/P_2_CS PAM	1.8 μM Palm_2_K-PEG_2_-M2_2–16_-PEG_2_-(KE)_4_ and 0.2 μM Pam_2_CS-M2_2–16_-PEG_2_-(KE)_4_

**Table 3:** In vivo vaccine treatment groups and dosages.

Experimental Group	Treatment
PBS	n/a
Orig PM	20 nmol M2_2–16_
Orig PM/Adj	20 nmol M2_2–16_ and 2.22 nmol Pam_2_CSK_4_
Orig PAM	20 nmol Palm_2_K-M2_2–16_-(KE)_4_
Orig PAM/Adj	20 nmol Palm_2_K-M2_2–16_-(KE)_4_ and 2.22 nmol Pam_2_CSK_4_
PP PAM	20 nmol Palm_2_K-PP-M2_2–16_-PP-(KE)_4_
ke PAM	20 nmol Palm_2_K-PEG_2_-M2_2–16_-(ke)_2_(KE)_2_
PEG_2_ PAM	20 nmol Palm_2_K-PEG_2_-M2_2–16_-PEG_2_-(KE)_4_
P_2_CS PAM	20 nmol Pam_2_CS-M2_2–16_-PEG_2_-(KE)_4_
PEG_2_ PAM/P_2_CS PAM	20 nmol Palm_2_K-PEG_2_-M2_2–16_-PEG_2_-(KE)_4_ and 2.22 nmol Pam_2_CS-M2_2–16_-PEG_2_-(KE)_4_

**Table 4:** Linker-containing PAs.

Abbreviation	Peptide/PA
	*Original PAM formulation:*
Orig PAM	Palm_2_K-M2_2–16_-(KE)_4_
	*New formulations:*
PP PAM	Palm_2_K-**PP**-M2_2–16_-**PP**-(KE)_4_
ke PAM	Palm_2_K-**PEG_2_**-M2_2–16_-**(ke)_2_**(KE)_2_
PEG_2_ PAM	Palm_2_K-**PEG_2_**-M2_2–16_-**PEG_2_**-(KE)_4_
P_2_CS PAM	**Pam_2_CS**-M2_2–16_-**PEG_2_**-(KE)_4_

New PAM formulations are listed with bolding and underlining to indicate changes from the original PAM formulation.

**Table 5:** Critical micelle concentrations of linker-containing peptide amphiphiles.

Formulation	Average CMC (μM) ± 1 Standard Deviation
Orig PM [15]	2.70 ± 1.60
Orig PAM [15]	0.15 ± 0.06
PP PAM	0.47 ± 0.14
ke PAM	0.07 ± 0.05
PEG_2_ PAM	0.14 ± 0.06
P_2_CS PAM	0.76 ± 0.20

**Table 6:** Linker-containing peptide amphiphile micelle sizes.

Formulation	Maximum Diameter (nm)	Minimum Diameter (nm)	Aspect Ratio
PP PAM	24 ± 17	13 ± 7	2.1 ± 1.1
ke PAM	22 ± 18	12 ± 9	1.9 ± 1.0
PEG_2_ PAM	22 ± 19	12 ± 8	1.9 ± 0.9
P_2_CS PAM	11 ± 4	8 ± 2	1.3 ± 0.4

## Data Availability

All data are contained within the article and Supplementary Materials.

## Supplementary Materials

The following supporting information can be downloaded at https://www.mdpi.com/article/10.3390/vaccines13040422/s1, Figure S1: Peptides and peptide amphiphiles were purified to greater than 90% purity using LC-MS; Figure S2: The gating strategy used for identifying activated bone-marrow-derived dendritic cells is illustrated above; Figure S3: Representative graphs of critical micelle concentrations showed that all peptide amphiphiles formed micelles; Figure S4: Circular dichroism of Pam2CSK4 aligns with a primarily random coil secondary structure as indicated by a minimum at slightly below 200 nm; Table S1. Statistical groups for each coating antigen in Figure 7; Table S2. Statistical groups for each vaccine in Figure 7b.
