# Supplementary material for: Adjuvant Templating Improves On-Target/Off-Target Antibody Ratio Better than Linker Addition for M2-Derived Peptide Amphiphile Micelle Vaccines
Source: Vaccines (Basel). 2025 Apr 17;13(4):422. doi: 10.3390/vaccines13040422 (PMC12031025; doi:10.3390/vaccines13040422)
Supplement: Supplementary file 1 [file vaccines-13-00422-s001.zip › vaccines-3529525-supplementary.pdf]

a **$M2_{2–16}$**

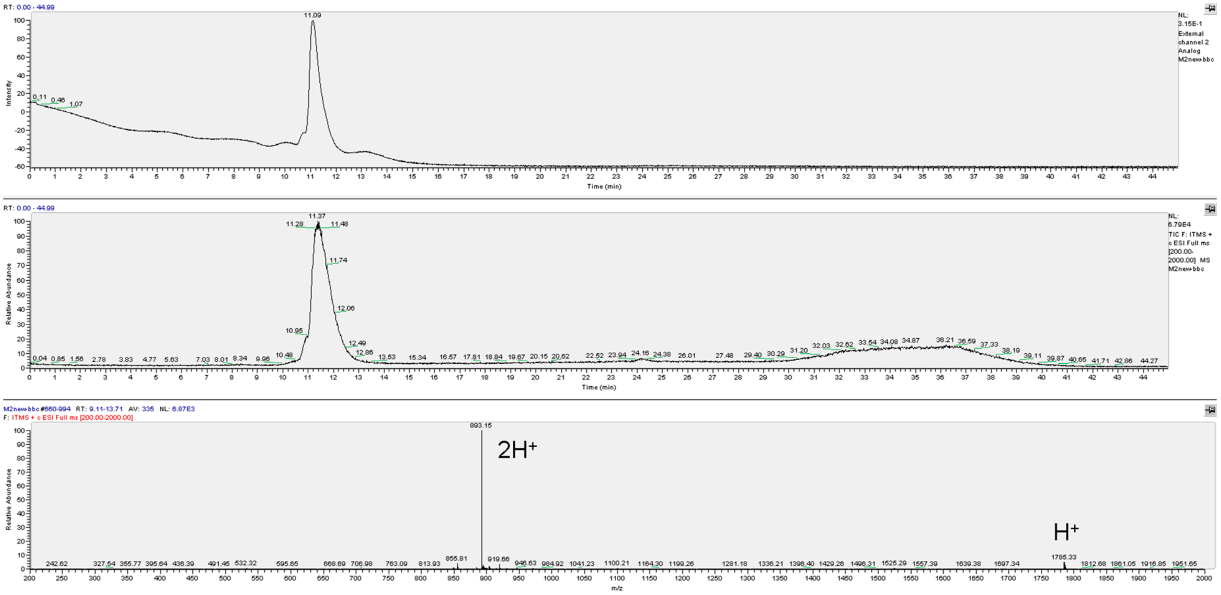

b **$Palm_2K$-$M2_{2–16}$-$(KE)_4$**

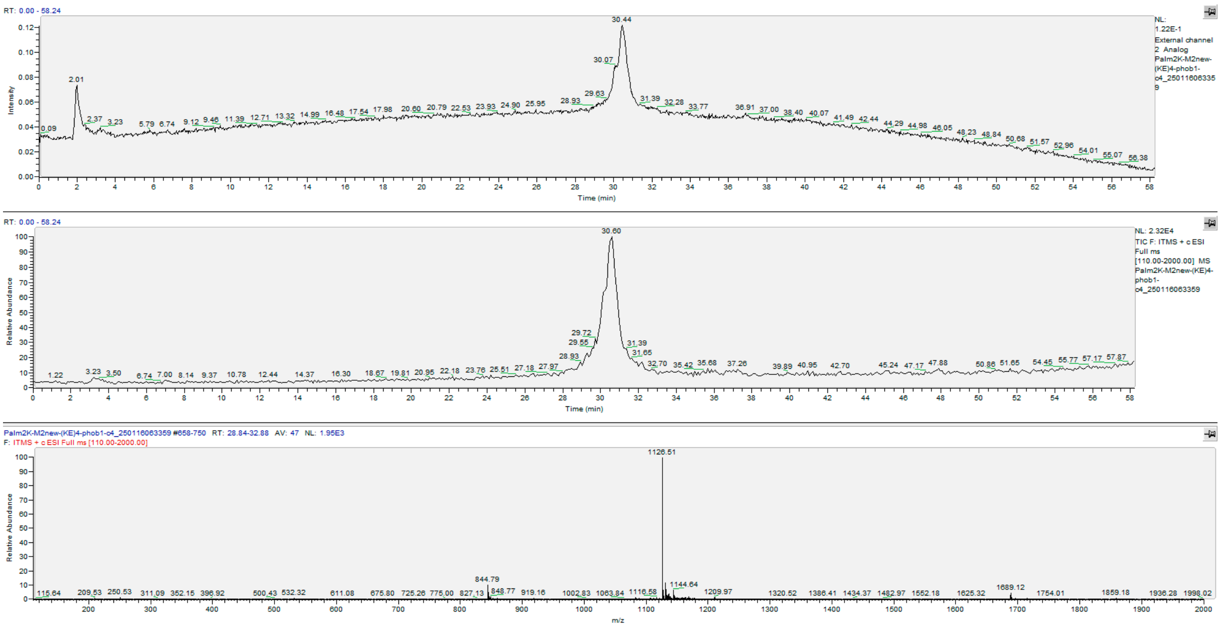

c **$M2_{10\text{–}16}$-$(KE)_4$**

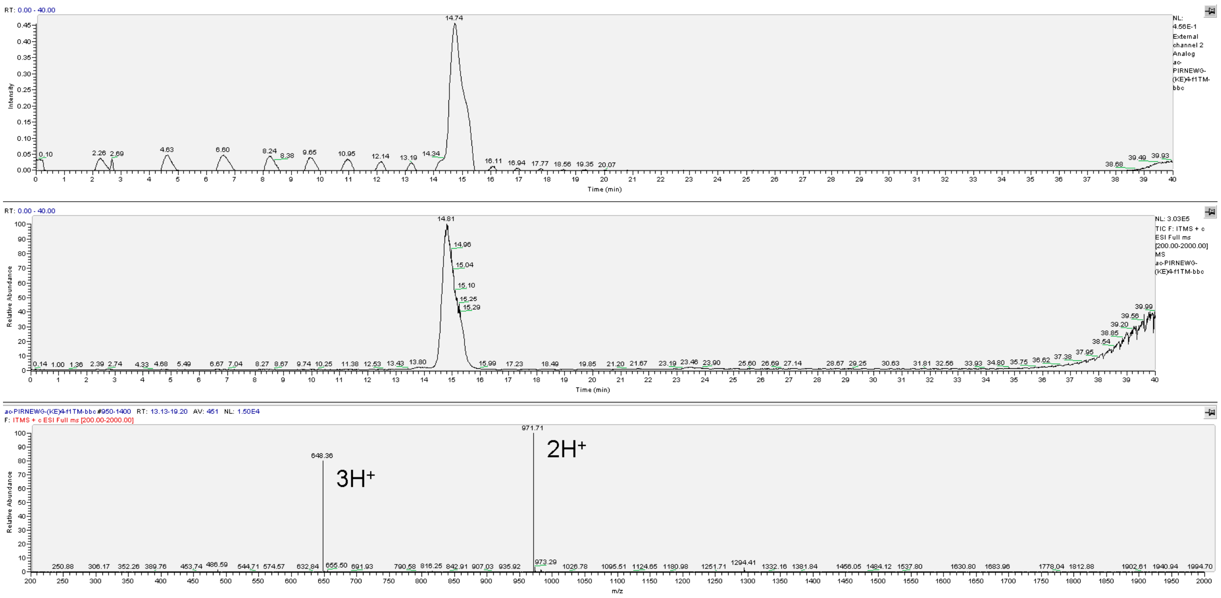

d **$(KE)_4$**

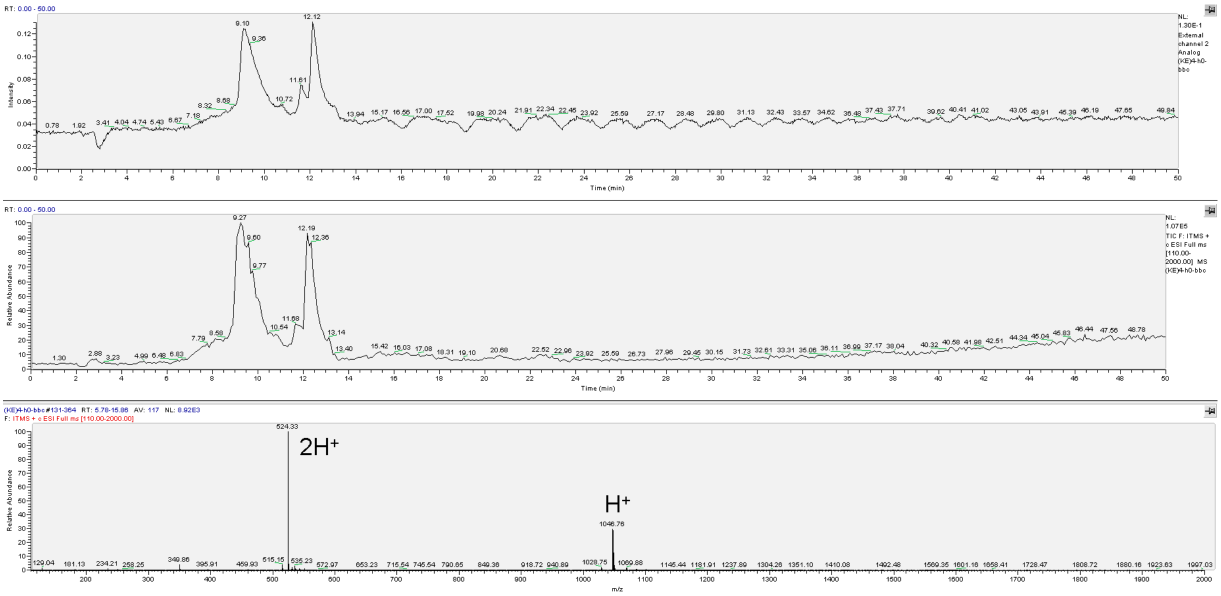

e Palm$_2$K-PP-M2$_{2–16}$-PP-(KE)$_4$

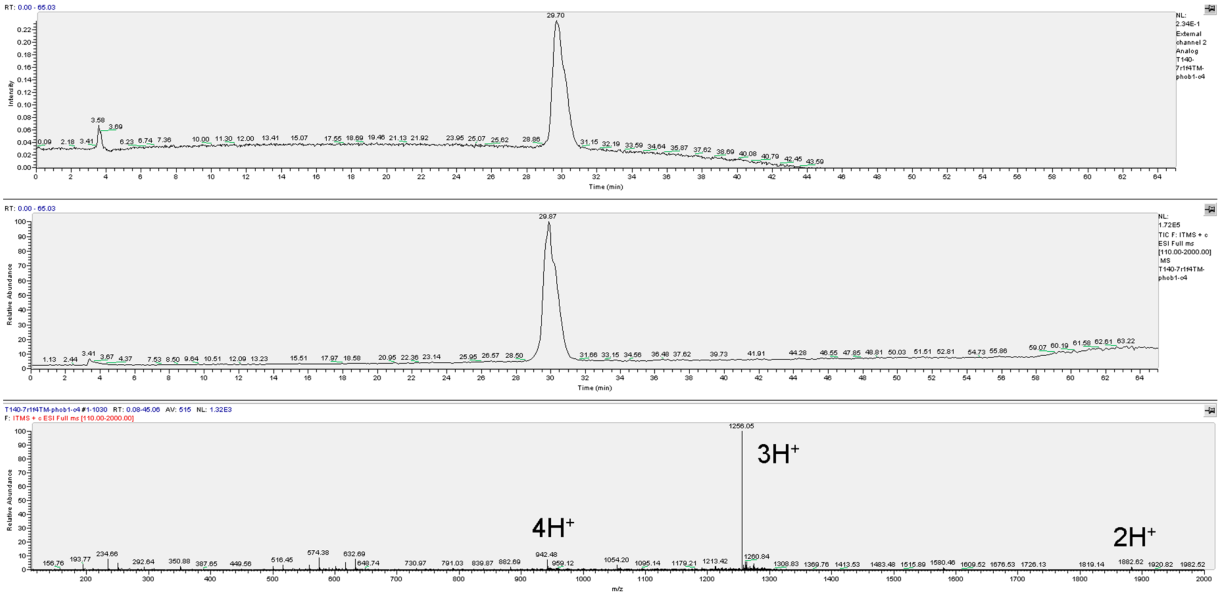

f Palm$_2$K-PEG$_2$-M2$_{2–16}$-(ke)$_2$(KE)$_2$

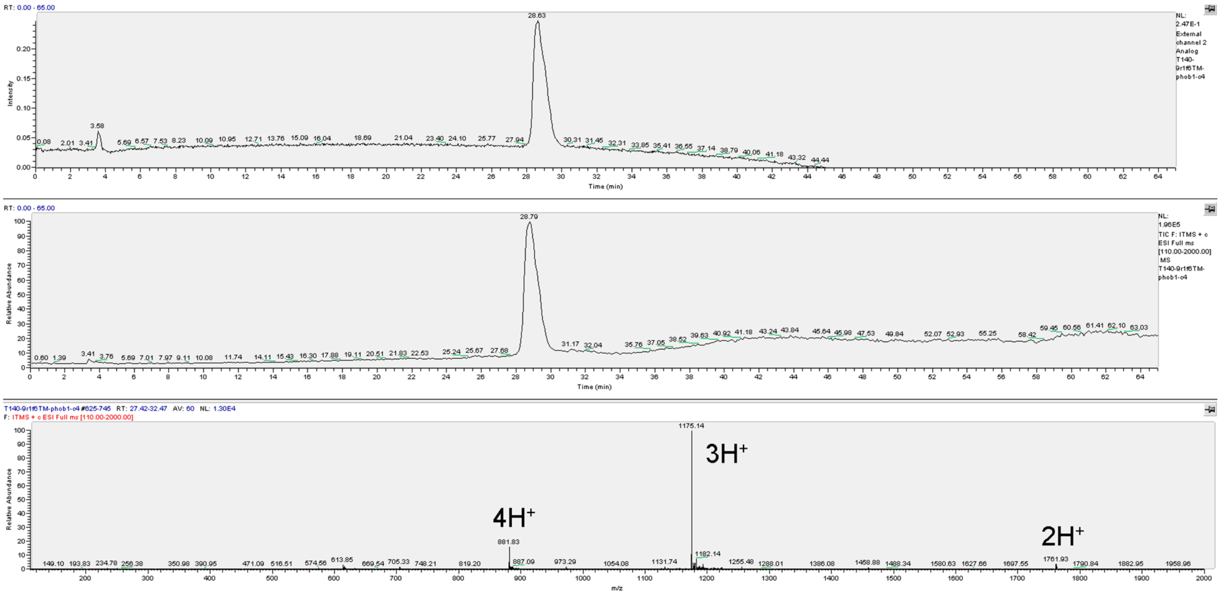

g Palm$_2$K-PEG$_2$-M2$_{2\text{–}16}$-PEG$_2$-(KE)$_4$

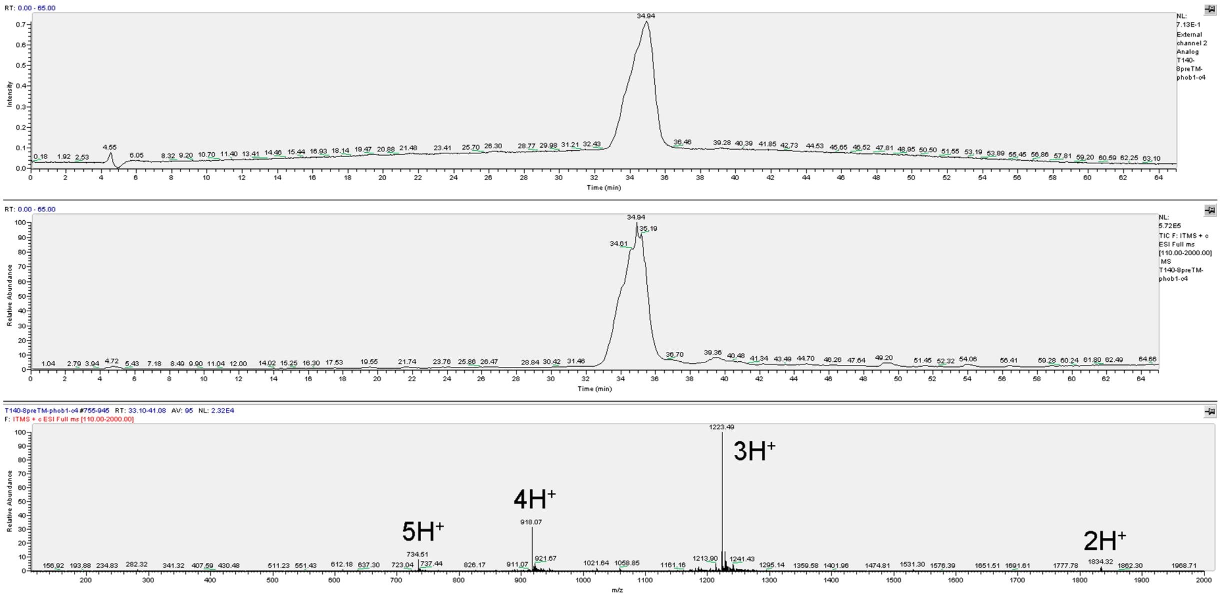

h Pam$_2$CS-M2$_{2\text{–}16}$-PEG$_2$-(KE)$_4$

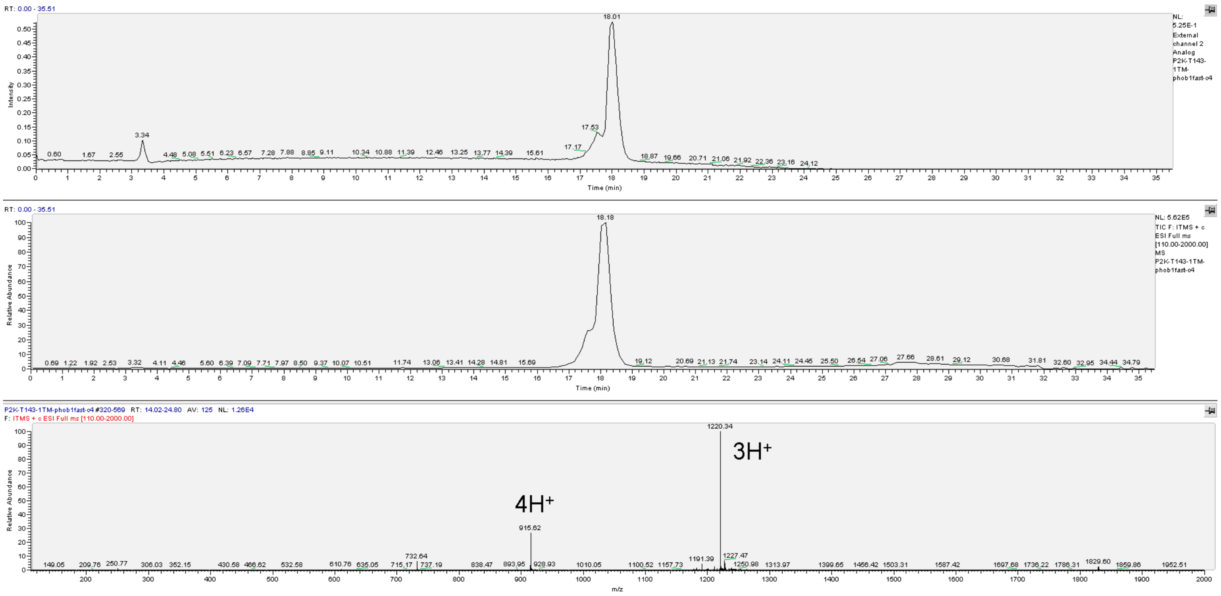

i M2$_{1–24}$

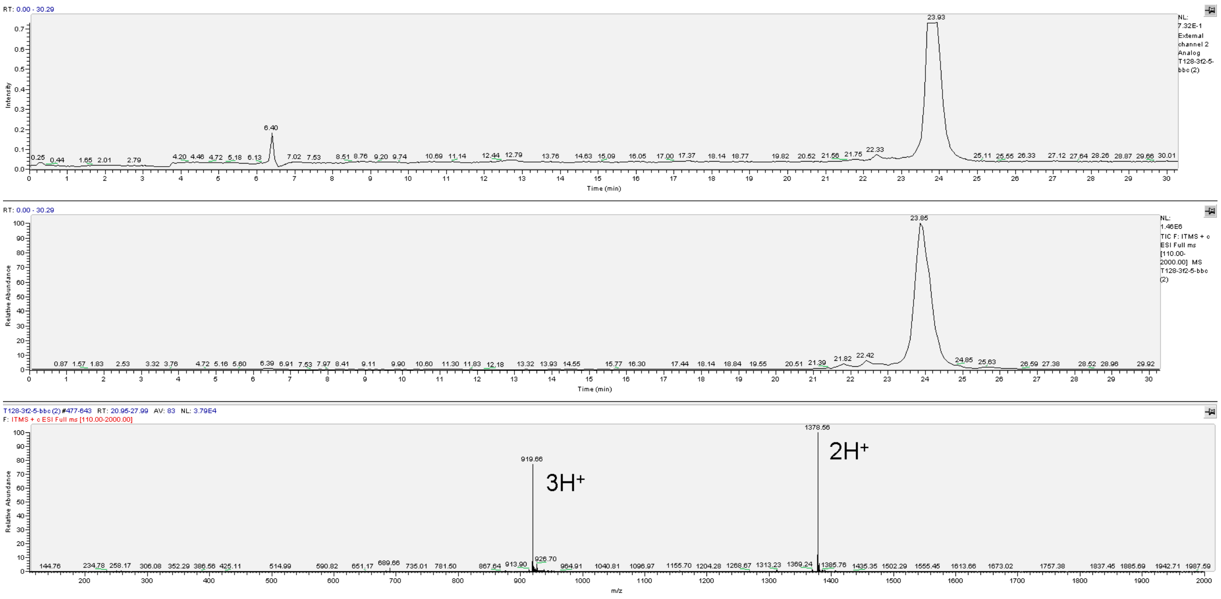

j Biotin-PEG$_2$-M2$_{2–16}$

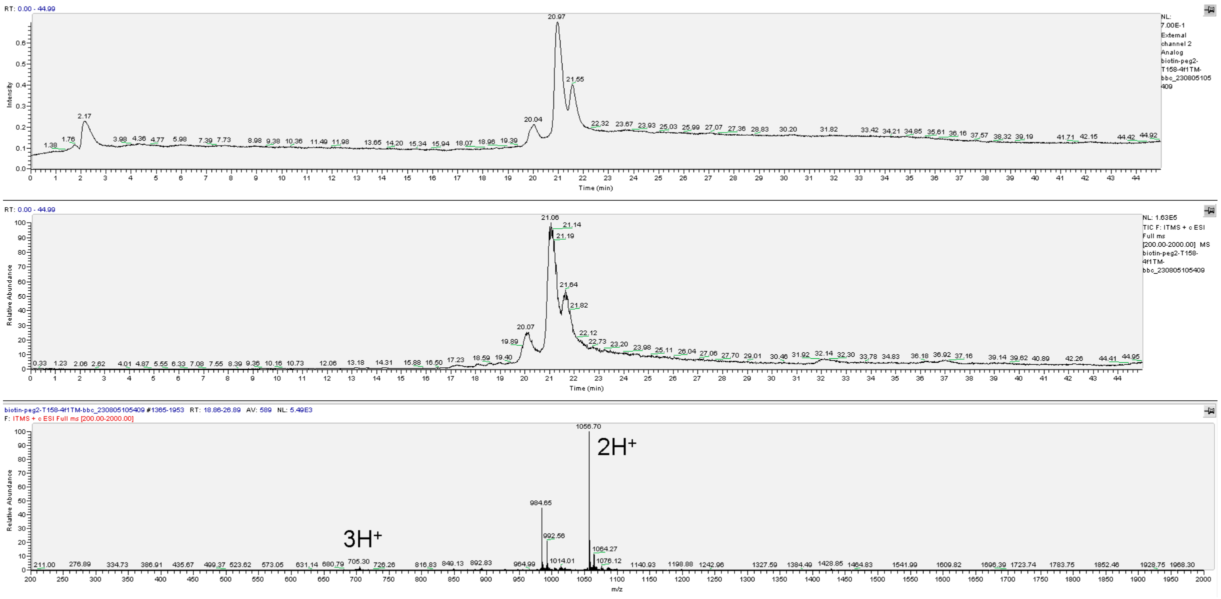

k Biotin-PEG$_2$-M2$_{2–16}$-PEG$_2$-(KE)$_4$

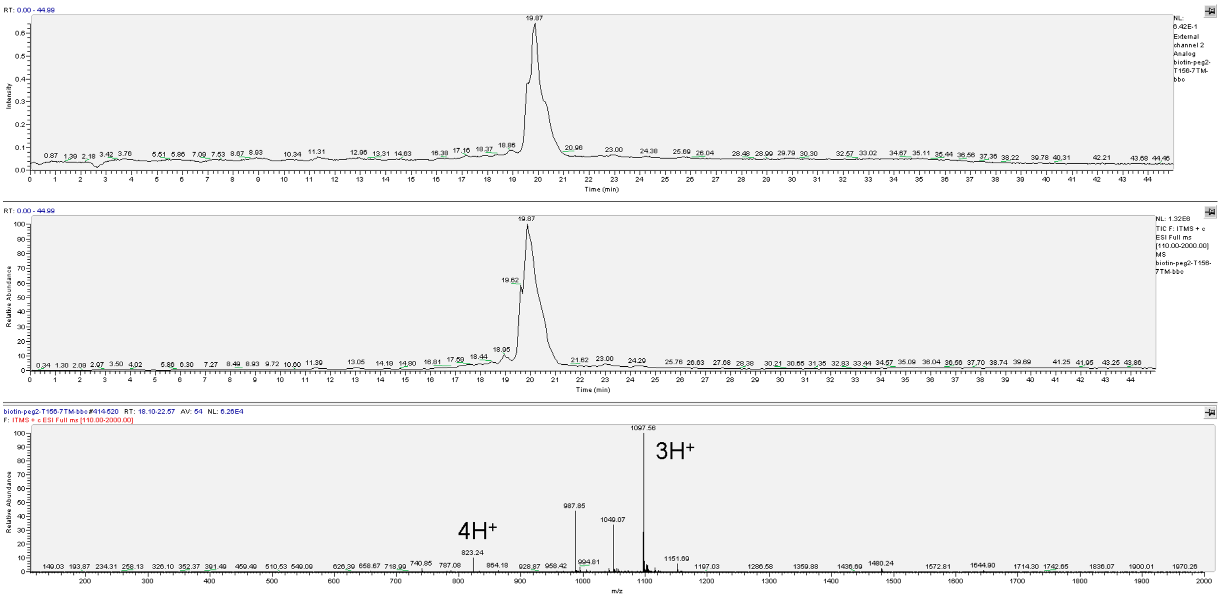

l Palm-RDRD-M2$_{2–16}$

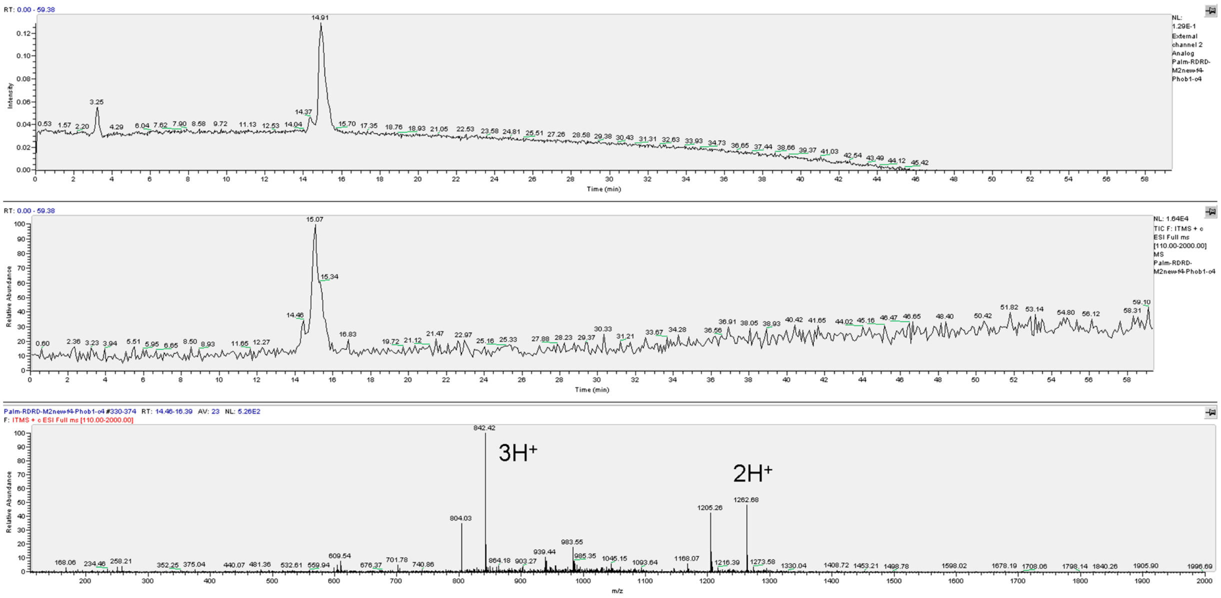

Figure S1. Peptides and peptide amphiphiles were purified to greater than 90% purity using LC-MS. Analyses are shown for (**a**) M2$_{2–16}$, (**b**) Palm$_2$K-M2$_{2–16}$-(KE)$_4$, (**c**) M2$_{10–16}$-(KE)$_4$, (**d**) (KE)$_4$, (**e**) Palm$_2$K-PP-M2$_{2–16}$-PP-(KE)$_4$, (**f**) Palm$_2$K-PEG$_2$-M2$_{2–16}$-(ke)$_2$(KE)$_2$, (**g**) Palm$_2$K-PEG$_2$-M2$_{2–16}$-PEG$_2$-(KE)$_4$, (**h**) Pam$_2$CS-M2$_{2–16}$-PEG$_2$-(KE)$_4$, (**i**) M2$_{1–24}$, (**j**) Biotin-PEG$_2$-M2$_{2–16}$, (**k**) Biotin-PEG$_2$-M2$_{2–16}$-PEG$_2$-(KE)$_4$, and (**l**) Palm-RDRD-M2$_{2–16}$. Panels from top to bottom for a given compound show the UV chromatogram, the total ion count chromatogram, and mass spectrum. On the mass spectrum, single through quintuple charge states are denoted, where applicable.

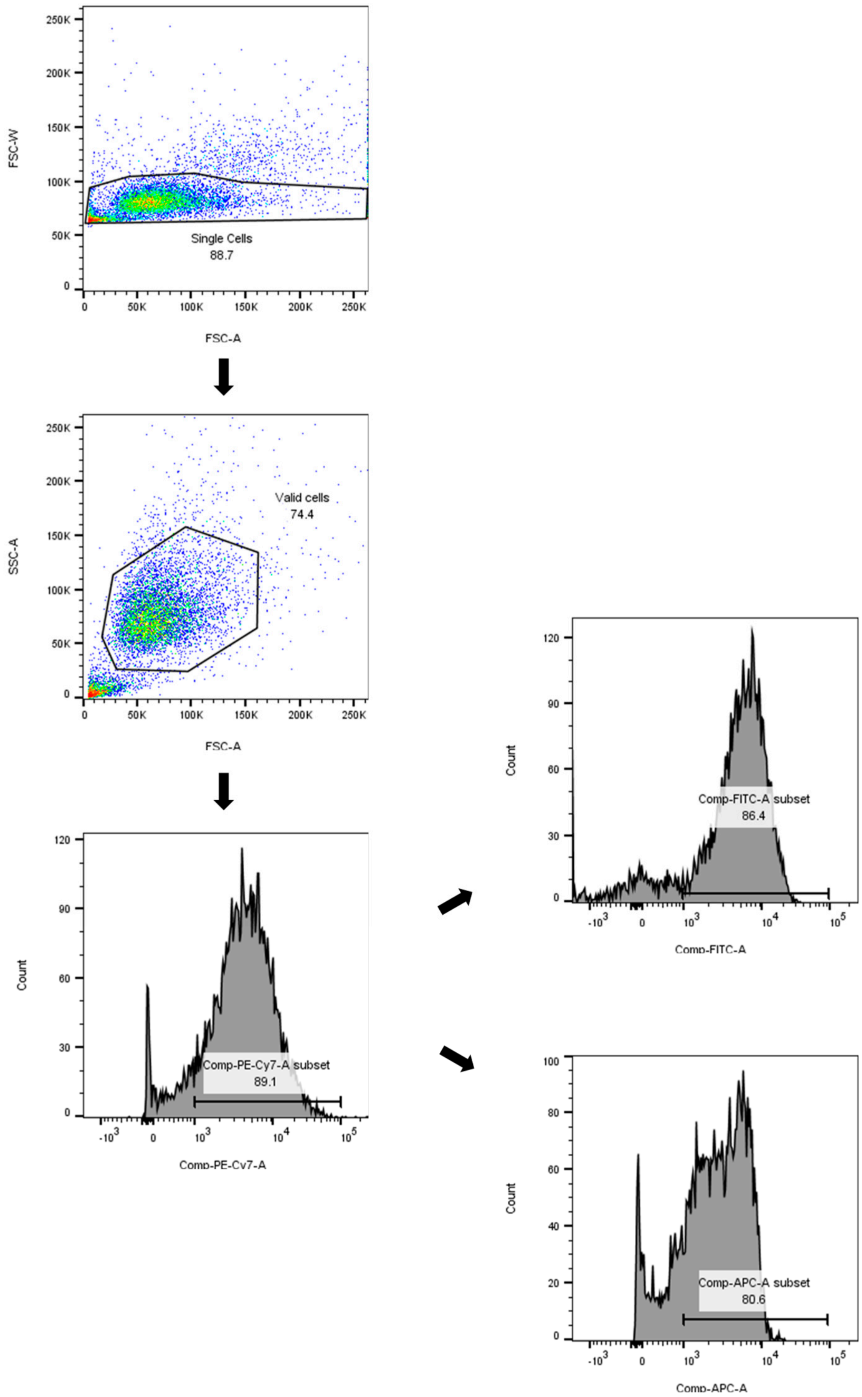

Figure S2. The gating strategy used for identifying activated bone marrow-derived dendritic cells is illustrated above. Single cells were isolated from cell aggregates and debris, first by forward scatter (FSC-A *versus* FSC-W), then by FSC *versus* SSC (side scatter). CD11c expression (PE-Cy7) was used to identify dendritic cells whereas cell activation was measured by CD40 (FITC) and MHC-II (APC) expression. FlowJo software was used to analyze the flow cytometry data.

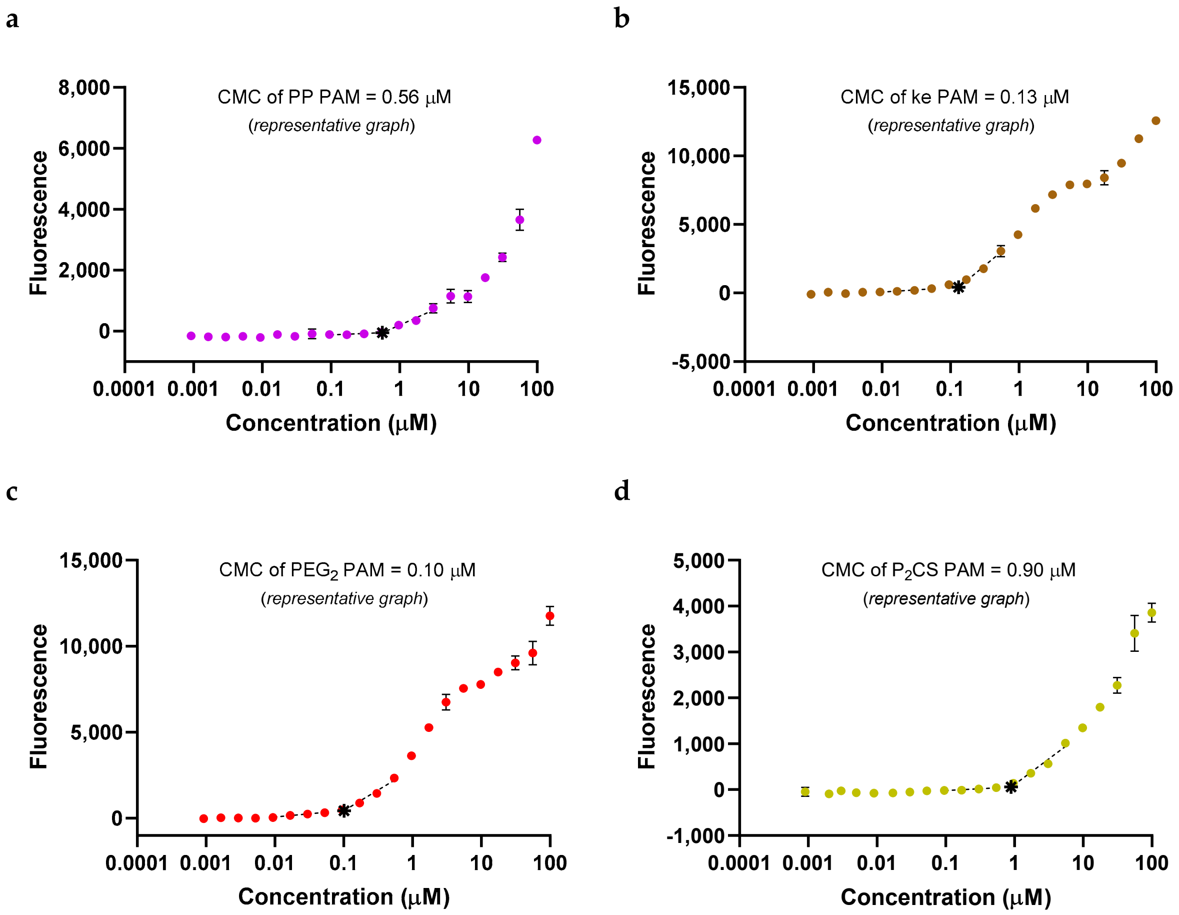

Figure S3. Representative graphs of the critical micelle concentration experiment showed that all peptide amphiphiles formed micelles (*i.e.*, had an identifiable jerk point). (**a**) PP PAMs micellized at 0.47 ± 0.14 μM, (**b**) ke PAMs micellized at 0.07 ± 0.05 μM, (**c**) PEG_2 PAMs micellized at 0.14 ± 0.06 μM, (d) and P_2CS PAMs micellized at 0.76 ± 0.20 μM. CMCs for the representative graphs are denoted by ∗.

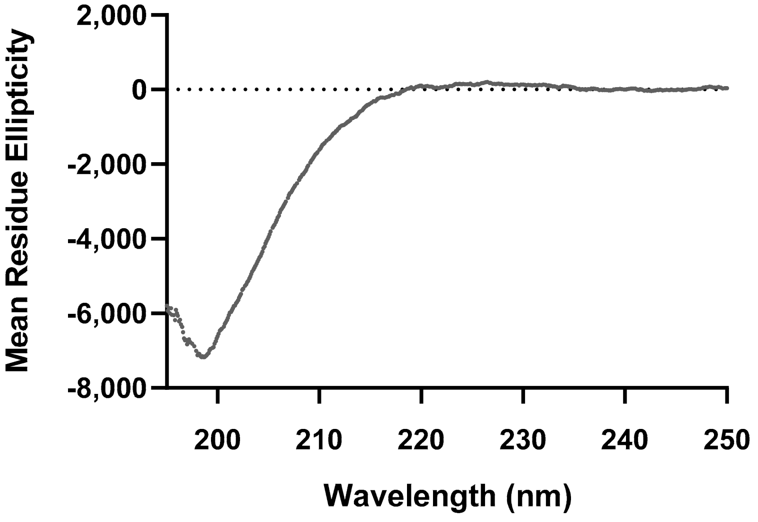

	**α-helix**	**β-sheet**	**random coil**
Pam_2CSK_4	0%	18.4 ± 1.7%	81.6 ± 1.7%

Figure S4. Circular dichroism of Pam_2CSK_4 reveals primarily random coil secondary structure, as indicated by a minimum at slightly less than 200 nm.

Table S1. Statistical groups for each coating antigen in Figure 7b

Vaccine	$M2_{2–16}$	Biotin-PEG_2-$M2_{2–16}$	$Palm_2K$-PEG_2-$M2_{2–16}$-PEG_2-$(KE)_4$	Biotin-PEG_2-$M2_{2–16}$-PEG_2-$(KE)_4$
PBS	A	A	A	A
Orig PM/Adj	C	BC	BC	DE
Orig PAM	AB	A	BC	CDE
Orig PAM/Adj	C	C	D	F
PP PAM	A	A	B	B
ke PAM	A	A	BC	BC
PEG_2 PAM	AB	A	BC	CD
P_2CS PAM	BC	B	CD	EF
PEG_2 PAM/P_2CS PAM	AB	A	BCD	DE

For each antigen coating (a given-colored bar), groups that possess different letters have statistically significant difference in mean ($p \leq 0.05$) whereas those that possess the same letter have similar means ($p > 0.05$). Statistical groups between antigen coatings (column-to-column) are unrelated.

Table S2. Statistical groups for each vaccine in Figure 7b

Coating Antigen	PBS	Orig PM/Adj	Orig PAM	Orig PAM/Adj	PP PAM	ke PAM	PEG_2 PAM	P_2CS PAM	PEG_2 PAM/P_2CS PAM
$M2_{2–16}$	Z	Z	Z	Z	YZ	Z	Y	Z	Z
Biotin-PEG_2-$M2_{2–16}$	Z	Z	Z	Z	Z	Z	Z	Z	Z
$Palm_2K$-PEG_2-$M2_{2–16}$-PEG_2-$(KE)_4$	Z	Z	Y	Y	X	Y	X	Y	Y
Biotin-PEG_2-$M2_{2–16}$-PEG_2-$(KE)_4$	Z	Z	Y	Y	XY	Y	X	Y	Y

For each antigen coating (a given cluster of bars), groups that possess different letters have statistically significant difference in mean ($p \leq 0.05$) whereas those that possess the same letter have similar means ($p > 0.05$). Statistical groups between antigen vaccines (column-to-column) are unrelated.
